# Development and Assessment of a Novel Whole-Gene-Based Targeted Next-Generation Sequencing Assay for Detecting the Susceptibility of Mycobacterium tuberculosis to 14 Drugs

**DOI:** 10.1128/spectrum.02605-22

**Published:** 2022-10-18

**Authors:** Sheng-Han Wu, Yu-Xin Xiao, Hseuh-Chien Hsiao, Ruwen Jou

**Affiliations:** a Tuberculosis Research Center, Centers for Disease Control, Ministry of Health and Welfare, Taipei, Taiwan; b Reference Laboratory of Mycobacteriology, Centers for Disease Control, Ministry of Health and Welfare, Taipei, Taiwan; Keck School of Medicine of the University of Southern California

**Keywords:** *Mycobacterium tuberculosis*, whole-genome sequencing, targeted NGS, drug susceptibility, heteroresistance, tuberculosis

## Abstract

Targeted next-generation sequencing (tNGS) has emerged as an alternative method for detecting drug-resistant tuberculosis (DR-TB). To provide comprehensive drug susceptibility information and to address mutations missed by available commercial molecular diagnostics, we developed and evaluated a tNGS panel with 22 whole-gene targets using the Ion Torrent platform to predict drug resistance to 14 drugs, namely, rifampicin (RIF), isoniazid (INH), ethambutol (EMB), pyrazinamide (PZA), moxifloxacin (MFX), levofloxacin (LFX), amikacin (AMK), capreomycin (CM), kanamycin (KM), streptomycin (SM), bedaquiline (BDQ), clofazimine (CFZ), linezolid (LZD), and delamanid (DLM). We selected 50 and 35 Mycobacterium tuberculosis isolates with various DR profiles as the training set and the challenge set, respectively. Comparative variant analyses of the DR genes were performed using Sanger sequencing and whole-genome sequencing (WGS). Phenotypic drug susceptibility testing (pDST) results were used as gold standards. Regarding the limit of detection, the tNGS assay detected 2.9 to 3.8% minority variants in 4% mutant mixtures. The sensitivity and specificity of tNGS were 97.0% (95% confidence interval [CI] = 93.1 to 98.7%) and 99.1% (95% CI = 97.7 to 99.7%), respectively. The concordance of tNGS with pDST was 98.5% (95% CI = 97.2 to 99.2%), which was comparable to that of WGS (98.7%, 95% CI = 97.4 to 99.3%) and better than that of Sanger sequencing (96.9%, 95% CI = 95.3 to 98.0%). The agreement between tNGS and pDST was almost perfect for RIF, INH, EMB, MFX, LFX, AMK, CM, KM, SM, BDQ, and LZD (kappa value = 0.807 to 1.000) and substantial for PZA (kappa value = 0.791). Our customized novel whole-gene-based tNGS panel is highly consistent with pDST and WGS for comprehensive and accurate prediction of drug resistance in a strengthened and streamlined DR-TB laboratory program.

**IMPORTANCE** We developed and validated a tNGS assay that was the first to target 22 whole genes instead of regions of drug resistance genes and comprehensively detected susceptibility to 14 anti-TB drugs, with great flexibility to include new or repurposed drugs. Notably, we demonstrated that our custom-designed Ion AmpliSeq TB research panel platform had high concordance with pDST and could significantly reduce turnaround time (by approximately 70%) to meet a clinically actionable time frame. Our tNGS assay is a promising DST solution for providing needed clinical information for precision medicine-guided therapies for DR-TB and allows the rollout of active pharmacovigilance.

## INTRODUCTION

Tuberculosis (TB) is an aerosol-transmitted bacterial infectious disease caused by Mycobacterium tuberculosis. The World Health Organization (WHO) End TB strategy prioritizes the early diagnosis of TB, including universal drug susceptibility testing (DST) (1). Drug-resistant TB (DR-TB) is a major challenge to the End TB strategy due to a gap between reported and estimated cases. In 2019, the WHO estimated that globally, 465,000 cases were DR-TB, of which only 206,030 (44.3%) and 177,099 (38.1%) were diagnosed and treated, respectively (2).

The current standard for comprehensive DR-TB diagnosis is culture-based DST. It usually takes months to obtain conventional DST results, which impacts patient outcomes and poses great risks to public health and challenges to global TB control. Rapid, accurate, and comprehensive diagnosis of DR-TB is crucial to provide personalized and precision medications for better treatment outcomes. WHO-endorsed genotypic DST (gDST) modalities, such as GenoType MTBDR*plus* and GenoType MTBDR*sl* (Bruker/Hain Lifescience, Nehren, Germany) (3, 4), Xpert MTB/RIF Ultra and Xpert MTB/XDR (Cepheid, Sunnyvale, CA, USA) (5, 6), and Truenat MTB-RIF-Dx (Molbio Diagnostics, Goa, India) (7), have limited detection capabilities, especially when rare or novel mutations are located outside detection regions of drug resistance-associated genes (8). Notably, whole-genome sequencing (WGS) and targeted next-generation sequencing (tNGS) have been used to predict drug resistance of TB since the WHO guideline was issued (9). With the continued expansion and reduced cost of NGS technology, WGS and tNGS could be adopted in TB control programs. In particular, tNGS can provide full-length sequence information with a large depth of coverage for targeted drug resistance genes, which is important for the prediction of heteroresistance (10).

Studies using tNGS for the prediction of TB drug resistance have shown crucial differences in the primer sets used in library preparation (11–15). In addition, the tested drug panel must be adjusted according to updated WHO treatment guidelines (16). Deeplex Myc-TB (Genoscreen, Lille, France) is a tNGS commercial assay kit that covers most drug resistance-related genes. Based on its design, the Deeplex Myc-TB kit can detect only mutations in the hot spot regions of drug resistance genes. The isoniazid (INH) resistance-associated gene mutation *fabG1* L203L cannot be detected (15). Furthermore, the primer sets of the Deeplex Myc-TB kit are not customized. The Ion AmpliSeq TB research panel (Thermo Fisher Scientific, Waltham, MA, USA), a tNGS commercial platform, has fewer targeted genes and can be customized.

To provide comprehensive information on susceptibility to 14 TB drugs recommended by the WHO treatment guidelines, we aimed to develop and standardize a tNGS assay on the Ion AmpliSeq TB platform as an alternative for detecting DR-TB.

## RESULTS

### Study design.

Table 1 summarizes commercial and customized primer sets for tNGS for the prediction of drug resistance. Two commercial assays were evaluated using an Ion AmpliSeq TB panel consisting of 8 whole-gene targets for 10 drugs (11) and the Deeplex Myc-TB assay targeting 6 whole-gene and hot spot regions of 12 genes for 14 drugs (15). In this study, we assessed a comprehensively designed tNGS assay with 22 whole-gene targets for 14 drugs, namely, rifampicin (RIF) (*rpoB*), INH (*katG*, *fabG1*, and *inhA*), ethambutol (EMB) (*embB*), pyrazinamide (PZA) (*pncA*), moxifloxacin (MFX) and levofloxacin (LFX) (*gyrA* and *gyrB*), amikacin (AMK), capreomycin (CM), kanamycin (KM), and streptomycin (SM) (*rrs*, *eis*, and *rpsL*), bedaquiline (BDQ) and clofazimine (CFZ) (*atpE*, *Rv0678*, *pepQ*, and *Rv1979c*), linezolid (LZD) (*rrl* and *rplC*), and delamanid (DLM) (*ddn*, *fgd1*, *fbiA*, *fbiB*, and *fbiC*) (Table 2).

**TABLE 1:** Summary and comparison of commercial and customized tNGS panels for prediction of drug resistance^*a*^

Drug(s)	Gene	Result with indicated tNGS panel (reference)					
		This study	Ion AmpliSeq TB (11)	Deeplex Myc-TB (15)	Tafess et al. (14)	Chan et al. (13)	Colman et al. (12)
RIF	*rpoB*	○	○	△	△	△	△
INH	*fabG1*	○		△^*b*^	△	△	
	*inhA*	○	○	△	△		△
	*katG*	○	○	△	△	△	△
	*ahpC*			△			
EMB	*embB*	○	○	△	△		
	*ubiA*				○		
PZA	*pncA*	○	○	○	○	○	
	*rpsA*				○		
MFX, LFX	*gyrA*	○	○	△	△	△	△
	*gyrB*	○		△	△		
AMK, CM, KM, SM	*rrs*	○		△	△	△	△
	*eis* promoter	○		○	○	○	○
	*eis*	○	○	△	△	△	△
	*tlyA*			○	○	△	
	*rpsL*	○	○	○	○	△	
	*whib7*				○		
BDQ, CFZ	*atpE*	○			○		
	*Rv0678*	○		○	○		
	*pepQ*	○					
	*Rv1979c*	○					
LZD	*rrl*	○		△	△		
	*rplC*	○		△	○		
DLM	*ddn*	○					
	*fgd1*	○					
	*fbiA*	○					
	*fbiB*	○					
	*fbiC*	○					
ETO	*ethA*			○			

a Detection of whole and hot spot regions of gene is indicated by circles and triangles, respectively. RIF, rifampicin; INH, isoniazid; EMB, ethambutol; PZA, pyrazinamide; MFX, moxifloxacin; LFX, levofloxacin; AMK, amikacin; CM, capreomycin; KM, kanamycin; SM, streptomycin; BDQ, bedaquiline; CFZ, clofazimine; LZD, linezolid; DLM, delamanid; ETO, ethionamide.

b Unable to detect *fabG1* L203L mutation.

**TABLE 2:** Information for Mycobacterium tuberculosis complex drug resistance genes for tNGS assay^*a*^

Gene	Genome positions	Gene positions	Codons	Avg depth (×)
*rpoB*	759735 to 763385	−72 to +61	−71 to full CDS	2,509
*katG*	2153865 to 2156228	−116 to +25	−116 to full CDS	1,940
*fabG1*	1673257 to 1674268	−183 to +86	−183 to full CDS	2,259
*inhA*	1674135 to 1675020	−67 to +10	−67 to full CDS	2,881
*embB*	4246468 to 4249822	−46 to +13	−46 to full CDS	1,231
*pncA*	2288670 to 2289361	−119 to +12	−119 to full CDS	2,101
*gyrA*	7263 to 9847	−39 to +30	−39 to full CDS	2,057
*gyrB*	5239 to 7391	−1 to +125	−1 to full CDS	2,478
*rrs*	1471830 to 1473462	−16 to +81	NA	2,097
*eis*	2714105 to 2715534	−201 to +20	−201 to full CDS	1,579
*rpsL*	781549 to 781983	−11 to +50	−11 to full CDS	2,586
*atpE*	1461005 to 1461310	−40 to +21	−40 to full CDS	3,132
*Rv0678*	778881 to 779541	−109 to +55	−109 to full CDS	2,079
*pepQ*	2859258 to 2860442	−23 to +43	−23 to full CDS	1,844
*rrl*	1473641 to 1476880	−17 to +86	NA	2,636
*rplC*	800714 to 801472	−95 to +11	−95 to full CDS	2,793
*Rv1979c*	2221706 to 2223283	−118 to +14	−118 to full CDS	2,608
*ddn*	3986732 to 3987332	−112 to +34	−112 to full CDS	1,994
*fgd1*	490636 to 491818	−147 to +26	−147 to full CDS	2,219
*fbiA*	3640397 to 3641598	−146 to +61	−146 to full CDS	1,520
*fbiB*	3641476 to 3642891	−59 to +11	−59 to full CDS	2,142
*fbiC*	1302803 to 1305527	−128 to +27	−128 to full CDS	1,776

a Positions of the reference sequences relative to the genome and genes of the M. tuberculosis H37Rv strain are indicated. Gene positions in promoter or 3′ region relative to the +1 or last nucleotide of coding sequences (CDS) are indicated by minus or plus signs, respectively. NA, not applicable for codons (positions outside a CDS or in rRNA gene region).

**FIG 1:**
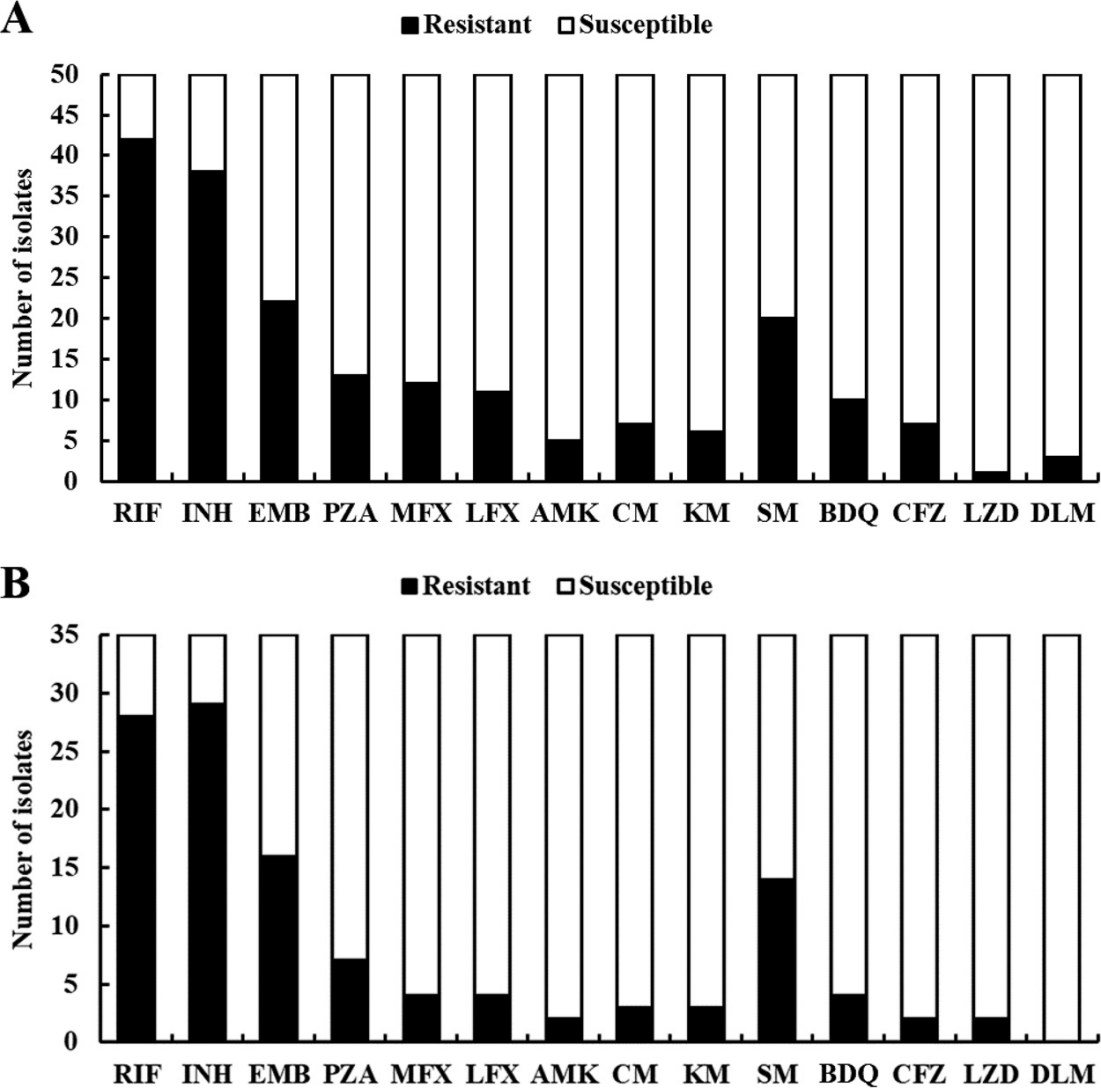
Drug resistance profile of the training isolates (*n* = 50) (A) and the challenge isolates (*n* = 35) (B) for evaluating the effectiveness of tNGS. RIF, rifampicin; INH, isoniazid; EMB, ethambutol; PZA, pyrazinamide; MFX, moxifloxacin; LFX, levofloxacin; AMK, amikacin; CM, capreomycin; KM, kanamycin; SM, streptomycin; BDQ, bedaquiline; CFZ, clofazimine; LZD, linezolid; DLM, delamanid.

A training set of 50 and a challenge set of 35 well-characterized Mycobacterium tuberculosis complex (MTBC) isolates were prepared for performance assessment using multiple methods, including tNGS, WGS, Sanger sequencing, and phenotypic DST (pDST). Figure 1A and Table S1 in the supplemental material show the drug resistance patterns of the training isolates, including resistance to RIF (*n* = 42 [84%]), INH (*n* = 38 [76%]), EMB (*n* = 23 [46%]), PZA (*n* = 13 [26%]), MFX (*n* = 12 [24%]), LFX (*n* = 11 [22%]), AMK (*n* = 5 [10%]), CM (*n* = 7 [14%]), KM (*n* = 6 [12%]), SM (*n* = 20 [40%]), BDQ (*n* = 10 [20%]), CFZ (*n* = 7 [14%]), LZD (*n* = 1 [2%]), and DLM (*n* = 3 [6%]). Based on the pDST results, 4% (2/50), 14% (7/50), 36% (18/50), 28% (14/50), 6% (3/50), and 12% (6/50) of cases were classified as pansusceptible TB, rifampicin-resistant TB (RR-TB), multidrug-resistant TB (MDR-TB), pre-extensively drug-resistant TB (pre-XDR-TB), extensively drug-resistant TB (XDR-TB), and others, respectively. Figure 1B and Table S2 show the drug resistance patterns of the challenge isolates, including resistance to RIF (*n* = 28 [80.0%]), INH (*n* = 29 [82.9%]), EMB (*n* = 16 [45.7%]), PZA (*n* = 8 [22.9%]), MFX (*n* = 4 [11.4%]), LFX (*n* = 4 [11.4%]), AMK (*n* = 2 [5.7%]), CM (*n* = 3 [8.6%]), KM (*n* = 3 [8.6%]), SM (*n* = 14 [40.0%]), BDQ (*n* = 4 [11.4%]), CFZ (*n* = 2 [5.7%]), LZD (*n* = 2 [5.7%]), and DLM (*n* = 0 [0.0%]). Based on the pDST results, 5.7% (2/35), 60.0% (21/35), 14.3% (5/35), and 20.0% (7/35) of cases were classified as RR-TB, MDR-TB, pre-XDR-TB, and others, respectively.

### Limit of detection (LOD).

**FIG 2:**
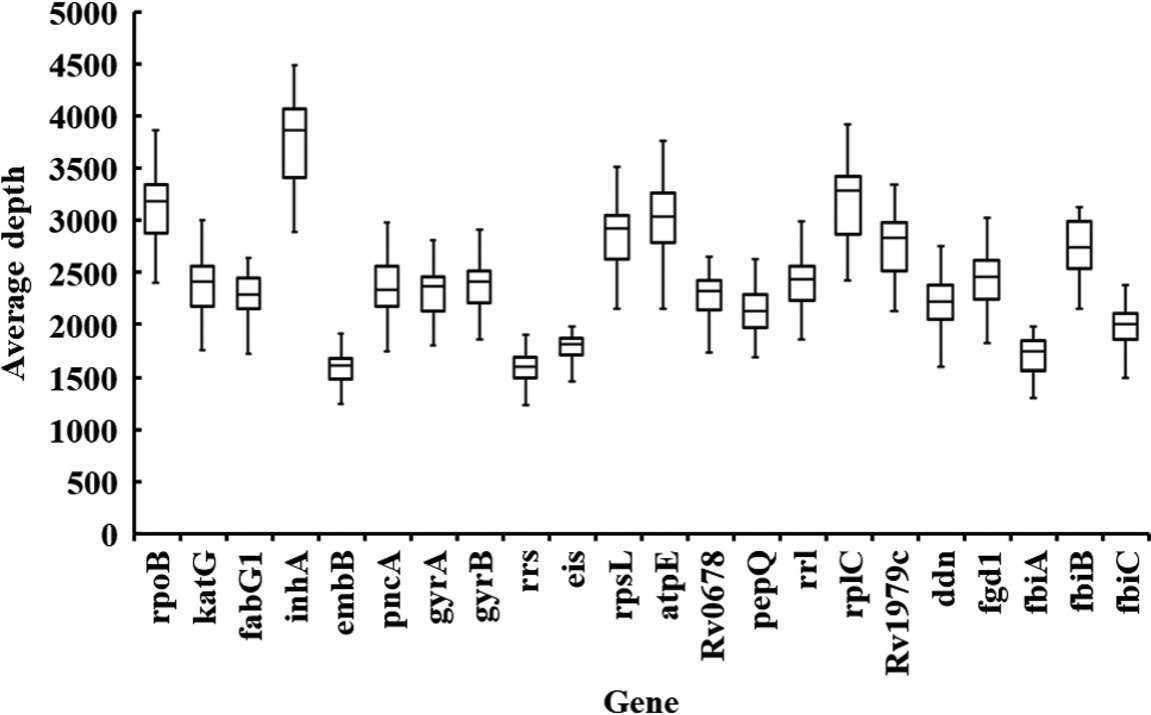
Sequencing depth at each drug resistance-associated gene by tNGS for heteroresistance.

The average depth of each gene obtained from the tNGS ranged from 1,578× to 3,743×, indicating the reliability of the variant proportion calculation (Fig. 2 and Table S3). The proportions obtained from the tNGS assay were consistent with the expected mixture ratios. The tNGS detected 3.8% ± 0.4% *rpoB*, 3.0% ± 0.4% *katG*, 3.5% ± 0.3% *embB*, 2.9% ± 0.4% *pncA*, and 3.3% ± 0.4% *gyrA* mutations in 4% mutant mixtures (Table 3). Sanger sequencing detected mutations in the *rpoB*, *katG*, *embB*, *pncA*, and *gyrA* genes from the mixture with at least 16% mutants (Table 3). pDST using the agar proportion method (APM) can detect 1% RIF-resistant, INH-resistant (0.2 μg/mL), and EMB-resistant isolates. However, pDST using a mycobacterial growth indicator tube (MGIT) can detect 8% PZA-resistant isolates and 32% MFX-resistant isolates.

**TABLE 3:** Detection of heteroresistance using tNGS and Sanger sequencing^*a*^

Drug	DST method	Finding for indicated mutant/H37Rv ratio (%)							
		100/0	32/68	16/84	8/92	4/96	2/98	1/99	0/100
RIF	pDST (μg/mL)								
	APM (1.0)	R	R	R	R	R	R	R	S
	gDST*_rpoB*								
	tNGS (VAF avg ± SD)	S531L (99.5 ± 0.2)	S531L (29.3 ± 1.1)	S531L (13.2 ± 0.2)	S531L (6.6 ± 0.5)	S531L (3.8 ± 0.4)	WT	WT	WT
	Sanger sequence	S531L	S531L mix WT	S531L mix WT	WT	WT	WT	WT	WT
INH	pDST (μg/mL)								
	APM (0.2)	R	R	R	R	R	R	R	S
	gDST*_katG*								
	tNGS (VAF avg ± SD)	S315T (99.8 ± 0.1)	S315T (24.8 ± 1.3)	S315T (11.3 ± 0.3)	S315T (5.2 ± 0.2)	S315T (3.0 ± 0.4)	WT	WT	WT
	Sanger sequence	S315T	S315T mix WT	S315T mix WT	WT	WT	WT	WT	WT
EMB	pDST (μg/mL)								
	APM (5.0)	R	R	R	R	R	R	R	S
	gDST*_embB*								
	tNGS (VAF avg ± SD)	D328Y (99.3 ± 0.2)	D328Y (26.6 ± 1.6)	D328Y (12.9 ± 0.2)	D328Y (6.3 ± 0.7)	D328Y (3.5 ± 0.3)	WT	WT	WT
	Sanger sequence	D328Y	D328Ymix WT	D328Y mix WT	WT	WT	WT	WT	WT
PZA	pDST (μg/mL)								
	MGIT (100)	R	R	R	R	S	S	S	S
	gDST*_pncA*								
	tNGS (VAF avg ± SD)	V155G (99.8 ± 0.1)	V155G (26.9 ± 1.6)	V155G (11.7 ± 0.4)	V155G (5.9 ± 0.7)	V155G (2.9 ± 0.4)	WT	WT	WT
	Sanger sequence	V155G	V155G mix WT	V155G mix WT	WT	WT	WT	WT	WT
MFX	pDST (μg/mL)								
	MGIT (0.25)	R	R	S	S	S	S	S	S
	gDST*_gyrA*								
	tNGS (VAF avg ± SD)	D94A (99.9 ± 0.1)	D94A (28.4 ± 1.4)	D94A (13.1 ± 1.5)	D94A (6.3 ± 0.8)	D94A (3.3 ± 0.4)	WT	WT	WT
	Sanger sequence	D94A	D94A mix WT	D94A mix WT	WT	WT	WT	WT	WT

a pDST, phenotypic drug susceptibility testing; gDST, genotypic drug susceptibility testing; tNGS, targeted NGS; APM, agar proportion method; VAF, variant allele frequency (percent); WT, wild type; R, resistant; S, susceptible.

### Performance of drug resistance prediction using the developed tNGS.

**FIG 3:**
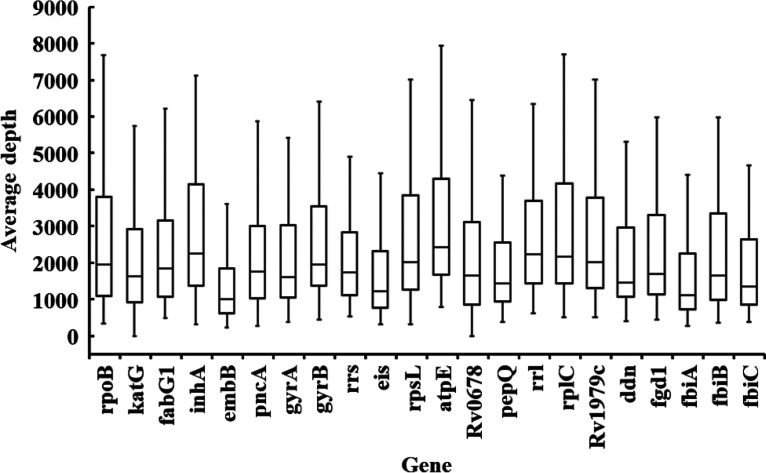
Sequencing depth at each drug resistance-associated gene on the total set of 85 isolates.

The average depth of each gene obtained from tNGS ranged from 1,231× to 3,132×, indicating the reliability of the variant proportion calculation (Table 2, Fig. 3, and Table S4). The category of resistance-associated mutations included in the tNGS was compared with the resistance mutations detected by genotypic assays and their associated phenotypic resistance. Validation of mutations was performed according to the WHO documentation (17).

For the 199 resistant phenotypes, the discordances between resistance in pDST and susceptibility in tNGS, WGS, and Sanger sequencing were 2.5% (5/199), 2.5% (5/199), and 8.5% (17/199), respectively (Table 4). Of these, 5 isolates with no mutation were phenotypically resistant to PZA (*n* = 2), MFX (*n* = 1), or CM (*n* = 2), 1 DLM-resistant isolate harbored an uncharacterized minority mutation (*fgd1* Ins 5 bp 35 to 36 frameshift mutation) detected only by tNGS, and 11 isolates carrying uncharacterized mutations detected by tNGS and WGS were phenotypically resistant to INH (*n* = 1 [*katG* L148Q)], EMB (*n* = 3 [*embB* g-6a, D814N, or H1002R]), or SM (*n* = 3 [*rrs* a13g, c594t, or Ins t1206–1207], *n* = 2 [*rrs* t16c], and *n* = 2 [*rrs* a514c]) (Table 4).

**TABLE 4:** Performance of gDST on the training isolates (*n* = 50)^*a*^

Drug	Method	pDST resistant				pDST susceptible				Performance (excluding uncharacterized variants)			
		gDST (no.)			Total	gDST (no.)			Total	Sensitivity, % (95% CI)	Specificity, % (95% CI)	Concordance, % (95% CI)	Agreement, kappa value (95% CI)
		R	S	U		R	S	U					
RIF	tNGS	42	0	0	42	2	6	0	8	100.0	75.0	96.0	0.834 (0.613–1.000)
	WGS	42	0	0	42	2	6	0	8	100.0	75.0	96.0	0.834 (0.613–1.000)
	Sanger	42	0	0	42	2	6	0	8	100.0	75.0	96.0	0.834 (0.613–1.000)
INH	tNGS	35	0	3	38	0	10	2	12	100.0	100.0	100.0	1.000 (1.000–1.000)
	WGS	35	0	3	38	0	10	2	12	100.0	100.0	100.0	1.000 (1.000–1.000)
	Sanger	35	1	2	38	0	11	1	12	97.2	100.0	97.9	0.943 (0.831–1.000)
EMB	tNGS	16	0	7	23	0	23	4	27	100.0	100.0	100.0	1.000 (1.000–1.000)
	WGS	16	0	7	23	0	23	4	27	100.0	100.0	100.0	1.000 (1.000–1.000)
	Sanger	16	3	4	23	0	26	1	27	84.2	100.0	93.3	0.860 (0.709–1.000)
PZA	tNGS	12	2	0	14	1	34	1	36	85.7	97.1	93.9	0.847 (0.679–1.000)
	WGS	12	2	0	14	0	35	1	36	85.7	100.0	95.9	0.896 (0.754–1.000)
	Sanger	12	2	0	14	0	35	1	36	85.7	100.0	95.9	0.896 (0.754–1.000)
MFX	tNGS	11	1	0	12	0	36	2	38	91.7	100.0	97.9	0.943 (0.832–1.000)
	WGS	11	1	0	12	0	36	2	38	91.7	100.0	97.9	0.943 (0.832–1.000)
	Sanger	11	1	0	12	0	37	1	38	91.7	100.0	98.0	0.943 (0.833–1.000)
LFX	tNGS	11	0	0	11	0	37	2	39	100.0	100.0	100.0	1.000 (1.000–1.000)
	WGS	11	0	0	11	0	37	2	39	100.0	100.0	100.0	1.000 (1.000–1.000)
	Sanger	11	0	0	11	0	38	1	39	100.0	100.0	100.0	1.000 (1.000–1.000)
AMK	tNGS	5	0	0	5	0	38	7	45	100.0	100.0	100.0	1.000 (1.000–1.000)
	WGS	5	0	0	5	0	38	7	45	100.0	100.0	100.0	1.000 (1.000–1.000)
	Sanger	5	0	0	5	0	42	3	45	100.0	100.0	100.0	1.000 (1.000–1.000)
CM	tNGS	5	2	0	7	0	36	7	43	71.4	100.0	95.3	0.807 (0.551–1.000)
	WGS	5	1	1	7	0	36	7	43	83.3	100.0	97.6	0.896 (0.694–1.000)
	Sanger	5	2	0	7	0	40	3	43	71.4	100.0	95.7	0.810 (0.556–1.000)
KM	tNGS	6	0	0	6	1	36	7	44	100.0	97.3	97.7	0.910 (0.735–1.000)
	WGS	6	0	0	6	1	36	7	44	100.0	97.3	97.7	0.910 (0.735–1.000)
	Sanger	6	0	0	6	1	41	2	44	100.0	97.6	97.9	0.911 (0.739–1.000)
SM	tNGS	15	0	5	20	0	30	0	30	100.0	100.0	100.0	1.000 (1.000–1.000)
	WGS	15	0	5	20	0	30	0	30	100.0	100.0	100.0	1.000 (1.000–1.000)
	Sanger	13	7	0	20	0	30	0	30	65.0	100.0	86.0	0.690 (0.488–0.893)
BDQ	tNGS	1	0	9	10	0	39	1	40	100.0	100.0	100.0	1.000 (1.000–1.000)
	WGS	1	0	9	10	0	40	0	40	100.0	100.0	100.0	1.000 (1.000–1.000)
	Sanger	1	0	9	10	0	40	0	40	100.0	100.0	100.0	1.000 (1.000–1.000)
CFZ	tNGS	0	0	7	7	0	37	6	43	NA	100.0	100.0	NA
	WGS	0	0	7	7	0	38	5	43	NA	100.0	100.0	NA
	Sanger	0	0	7	7	0	38	5	43	NA	100.0	100.0	NA
LZD	tNGS	1	0	0	1	0	39	10	49	100.0	100.0	100.0	1.000 (1.000–1.000)
	WGS	1	0	0	1	0	39	10	49	100.0	100.0	100.0	1.000 (1.000–1.000)
	Sanger	1	0	0	1	0	44	5	49	100.0	100.0	100.0	1.000 (1.000–1.000)
DLM	tNGS	0	0	3	3	0	33	14	47	NA	100.0	100.0	NA
	WGS	0	1	2	3	0	37	10	47	NA	100.0	97.4	NA
	Sanger	0	1	2	3	0	37	10	47	NA	100.0	97.4	NA
Total	tNGS	160	5	34	199	4	434	63	501	97.0 (93.1–98.7)	99.1 (97.7–99.7)	98.5 (97.2–99.2)	0.962 (0.938–0.987)
	WGS	160	5	34	199	3	440	58	501	97.0 (93.1–98.7)	99.3 (98.0–99.8)	98.7 (97.4–99.3)	0.967 (0.944–0.990)
	Sanger	158	17	24	199	3	465	33	501	90.3 (85.0–93.9)	99.4 (98.1–99.8)	96.9 (95.3–98.0)	0.920 (0.885–0.954)

a Sanger, Sanger sequencing; WGS, whole-genome sequencing; R, detection of resistance-associated mutation; S, detection of mutations known to not be associated with resistance (phylogenetic marker or synonymous mutation) or no mutation detected; U, detection of at least one novel nonsynonymous mutation; NA, not available; CI, confidence interval.

For the 501 susceptible phenotypes, the discordances between susceptibility in pDST and resistance in tNGS, WGS, and Sanger sequencing were 0.8% (4/501), 0.6% (3/501), and 0.6% (3/501), respectively (Table 4). Of these isolates, 3 isolates harboring disputed mutations or resistance-associated mutations were phenotypically susceptible to RIF (*n* = 1 [*rpoB* L511P] and *n* = 1 [*rpoB* L533P]) or KM (*n* = 1 [*eis* c-12t]); 1 isolate carrying a minority resistance-associated mutation (*pncA* H71Y) detected by tNGS was phenotypically susceptible to PZA (Table 4).

For the 700 resistant and susceptible phenotypes, the percentages of uncharacterized mutations predicted by tNGS, WGS, and Sanger sequencing were 13.9% (97/700), 13.1% (92/700), and 8.1% (57/700), respectively (Table 4). The performance of phenotypes predicted by gDST is shown in Table 4. The sensitivities of tNGS, WGS, and Sanger sequencing were 97.0% (71.4 to 100.0%), 97.0% (83.3 to 100.0%), and 90.2% (65.0 to 100.0%), respectively, while specificities were 99.1% (75.0 to 100.0%), 99.3% (75.0 to 100.0%), and 99.4% (75.0 to 100.0%), respectively (Table 4). The concordances of tNGS, WGS, and Sanger sequencing with pDST were 98.5% (93.9 to 100.0%), 98.7% (95.9 to 100.0%), and 96.9% (86.0 to 100.0%), respectively. The agreement between tNGS and pDST showed kappa values of 1.000 for INH, EMB, LFX, AMK, SM, BDQ, and LZD (almost perfect) and 0.807 to 0.943 for RIF, PZA, MFX, CM, and KM (almost perfect) (Table 4). However, the agreement for CFZ and DLM could not be estimated because isolates determined to be resistant by pDST harbored uncharacterized mutations (Table 4).

### Validation of tNGS for predicting drug resistance.

For the 119 resistant phenotypes, the discordance between resistance in pDST and susceptibility in tNGS was 3.4% (4/119) (Table 5). The 4 isolates with no mutation were phenotypically resistant to EMB (*n* = 1), CM (*n* = 1), KM (*n* = 1), and SM (*n* = 1).

**TABLE 5:** Validation of gDST on the challenge isolates (*n* = 35)

Drug	Method	pDST resistant				pDST susceptible				Performance (excluding uncharacterized variants)			
		gDST (N)			Total	gDST (N)			Total	Sensitivity, % (95% CI)	Specificity, % (95% CI)	Concordance, % (95% CI)	Agreement, kappa value (95% CI)
		R	S	U		R	S	U					
RIF	tNGS	28	0	0	28	0	5	2	7	100.0	100.0	100.0	1.000 (1.000–1.000)
	WGS	28	0	0	28	0	5	2	7	100.0	100.0	100.0	1.000 (1.000–1.000)
	Sanger	28	0	0	28	0	5	2	7	100.0	100.0	100.0	1.000 (1.000–1.000)
INH	tNGS	24	0	5	29	0	4	2	6	100.0	100.0	100.0	1.000 (1.000–1.000)
	WGS	24	0	5	29	0	4	2	6	100.0	100.0	100.0	1.000 (1.000–1.000)
	Sanger	24	1	4	29	0	4	2	6	96.0	100.0	96.6	0.869 (0.618–1.000)
EMB	tNGS	13	1	2	16	1	15	3	19	92.9	93.8	93.3	0.866 (0.687–1.000)
	WGS	13	1	2	16	1	16	2	19	92.9	94.1	93.5	0.870 (0.695–1.000)
	Sanger	13	1	2	16	1	17	1	19	92.9	94.4	93.8	0.873 (0.703–1.000)
PZA	tNGS	5	0	3	8	0	27	0	27	100.0	100.0	100.0	1.000 (1.000–1.000)
	WGS	5	0	3	8	0	27	0	27	100.0	100.0	100.0	1.000 (1.000–1.000)
	Sanger	5	0	3	8	0	27	0	27	100.0	100.0	100.0	1.000 (1.000–1.000)
MFX	tNGS	4	0	0	4	0	27	4	31	100.0	100.0	100.0	1.000 (1.000–1.000)
	WGS	4	0	0	4	0	27	4	31	100.0	100.0	100.0	1.000 (1.000–1.000)
	Sanger	4	0	0	4	0	29	2	31	100.0	100.0	100.0	1.000 (1.000–1.000)
LFX	tNGS	4	0	0	4	0	27	4	31	100.0	100.0	100.0	1.000 (1.000–1.000)
	WGS	4	0	0	4	0	27	4	31	100.0	100.0	100.0	1.000 (1.000–1.000)
	Sanger	4	0	0	4	0	29	2	31	100.0	100.0	100.0	1.000 (1.000–1.000)
AMK	tNGS	2	0	0	2	0	31	2	33	100.0	100.0	100.0	1.000 (1.000–1.000)
	WGS	2	0	0	2	0	31	2	33	100.0	100.0	100.0	1.000 (1.000–1.000)
	Sanger	2	0	0	2	0	32	1	33	100.0	100.0	100.0	1.000 (1.000–1.000)
CM	tNGS	2	1	0	3	0	30	2	32	66.7	100.0	97.0	0.784 (0.378–1.000)
	WGS	2	0	1	3	0	30	2	32	100.0	100.0	100.0	1.000 (1.000–1.000)
	Sanger	2	1	0	3	0	31	1	32	66.7	100.0	97.1	0.785 (0.379–1.000)
KM	tNGS	2	1	0	3	1	29	2	32	66.7	96.7	93.9	0.633 (0.165–1.000)
	WGS	2	1	0	3	1	29	2	32	66.7	96.7	93.9	0.633 (0.165–1.000)
	Sanger	2	1	0	3	1	31	0	32	66.7	96.9	94.3	0.635 (0.169–1.000)
SM	tNGS	13	1	0	14	0	20	1	21	92.9	100.0	97.1	0.939 (0.820–1.000)
	WGS	13	0	1	14	0	20	1	21	100.0	100.0	100.0	1.000 (1.000–1.000)
	Sanger	10	4	0	14	0	21	0	21	71.4	100.0	88.6	0.750 (0.527–0.973)
BDQ	tNGS	1	0	3	4	0	29	2	31	100.0	100.0	100.0	1.000 (1.000–1.000)
	WGS	1	0	3	4	0	29	2	31	100.0	100.0	100.0	1.000 (1.000–1.000)
	Sanger	1	0	3	4	0	29	2	31	100.0	100.0	100.0	1.000 (1.000–1.000)
CFZ	tNGS	0	0	2	2	0	29	4	33	NA	100.0	100.0	NA
	WGS	0	0	2	2	0	29	4	33	NA	100.0	100.0	NA
	Sanger	0	0	2	2	0	29	4	33	NA	100.0	100.0	NA
LZD	tNGS	2	0	0	2	0	28	5	33	100.0	100.0	100.0	1.000 (1.000–1.000)
	WGS	2	0	0	2	0	28	5	33	100.0	100.0	100.0	1.000 (1.000–1.000)
	Sanger	2	0	0	2	0	31	2	33	100.0	100.0	100.0	1.000 (1.000–1.000)
DLM	tNGS	0	0	0	0	0	31	4	35	NA	100.0	100.0	NA
	WGS	0	0	0	0	0	31	4	35	NA	100.0	100.0	NA
	Sanger	0	0	0	0	0	31	4	35	NA	100.0	100.0	NA
Total	tNGS	100	4	15	119	2	332	37	371	96.2 (90.5–98.5)	99.4 (97.8–99.8)	98.6 (97.0–99.4)	0.962 (0.932–0.992)
	WGS	100	2	17	119	2	333	36	371	98.0 (93.1–99.5)	99.4 (97.9–99.8)	99.1 (97.7–99.6)	0.974 (0.949–0.999)
	Sanger	97	8	14	119	2	346	23	371	92.4 (85.7–96.1)	99.4 (97.9–99.8)	97.8 (96.0–98.8)	0.937 (0.898–0.976)

For the 371 susceptible phenotypes, the discordance between susceptibility in pDST and resistance in tNGS was 0.5% (2/371) (Table 5). Of these isolates, 1 carrying a resistance-associated mutation (*embB* M306V) was phenotypically susceptible to EMB; the other isolate, harboring a low-level resistance mutation (*eis* c-12t), was phenotypically susceptible to KM (Table 5).

Overall, the sensitivity, specificity, and concordance of phenotypes predicted by tNGS were 96.2% (66.7 to 100.0%), 99.4% (93.8 to 100.0%), and 98.6% (93.3 to 100.0%), respectively (Table 5). The agreement between tNGS and pDST showed kappa values of 1.000 for RIF, INH, PZA, MFX, LFX, AMK, BDQ, and LZD (almost perfect), 0.866 for EMB (almost perfect), 0.939 for SM (almost perfect), 0.784 for CM (substantial), and 0.633 for KM (substantial) (Table 5).

### Investigating uncharacterized variants.

Table S5 shows 47 and 100 uncharacterized novel or rare variants with either resistant or susceptible phenotypes detected by tNGS (Tables 4 and 5 and Table S5). Isolates with novel or rare variants identified in genes conferring resistance to MFX/LFX, LZD, and second-line injectable drugs (SLIDs) were phenotypically susceptible. Nevertheless, the association between uncharacterized mutations and phenotypic drug resistance in M. tuberculosis needs to be validated.

## DISCUSSION

WHO TB guidelines emphasize the importance of DST before treatment and recommend the rapid uptake of new advanced technologies. We developed and validated a tNGS assay that was the first to target whole genes instead of regions of drug resistance genes and comprehensively detected susceptibility to anti-TB drugs with great flexibility to include new or repurpose drugs. Notably, compared to pDST, our novel tNGS assay had high concordance, with an overall kappa value of 0.962 (0.807 to 1.000), and helped achieve a significant reduction (of approximately 70%) in turnaround time to meet a clinically actionable time frame. This study demonstrated that our custom-designed Ion AmpliSeq TB research panel platform detected susceptibility to 14 drugs targeting 22 genes with a consistency of 98.5% (93.9 to 100.0%) with pDST (Table 4), in contrast to the original commercialized platform, which can detect susceptibility to 10 anti-TB drugs targeting 8 genes, with a consistency with pDST of 94.8% (90.0% to 100.0%) (11). However, the Deeplex Myc-TB platform detects susceptibility to 14 anti-TB drugs targeting 18 genes, and its consistency with pDST is 95.5% (85.7% to 100.0%) (15). Our tNGS assay is a promising DST solution for providing needed clinical information for precision medicine-guided therapies for DR-TB and allows the rollout of active pharmacovigilance. Fortunately, only a few patients were infected by isolates that were phenotypically resistant to BDQ, CFZ, LZD, or DLM in Taiwan. Therefore, we included 4 WHO proficiency test (WHO-PT) isolates in the training set. Nevertheless, we did observe uncharacterized mutations in phenotypically resistant isolates in the training set. The correlation between these mutations and drug resistance still needed to be verified (Table 4).

Current WHO-endorsed molecular diagnostics for the detection of DR-TB can be used to detect only a limited number of target gene regions and are not ideal for the detection of novel mutations located outside the targeted region or phenotypic resistance conferred by mutations across large gene regions, such as *pncA* for PZA, even *atpE* (18), *Rv0678* (19), and *pepQ* (20) for the novel drug BDQ and *ddn*, *fgd1*, *fbiA*, *fbiB*, and *fbiC* (21) for the novel drug DLM, and *Rv0678* (19) and *Rv1979c* (22) for the repurposed drug CFZ and *rrl* (23) and *rplC* (24) for the repurposed drug LZD. Consequently, discordant results between DST methods were observed. To guide optimal dosage regimen determinations, discordant diagnostic results must be resolved, and treatment effectiveness must be monitored, meaning that pDST cannot be completely eliminated by gDST.

Furthermore, the novel panel of tNGS could be extended to predict drug resistance to rifabutin (RFB) and ethionamide (ETO), as well as others beyond the 14 drugs (Table S6). RIF and RFB, semisynthetic derivatives of rifamycin S, are rifamycins (25). RFB is recommended for treating patients with TB and HIV coinfection because it has fewer drug interactions with protease inhibitor drugs (26) and for treating RFB-susceptible MDR-TB patients (27). ETO is one of the group C drugs for adult MDR-TB treatment (16) and is the most commonly used second-line oral drug for childhood TB treatment (28). In particular, ETO can be used for the treatment of tuberculous meningitis and miliary TB due to good cerebrospinal fluid (CSF) penetration (29).

Resistance to RFB among members of the MTBC is mainly associated with mutations within the RIF resistance-determining region (RRDR) in the *rpoB* gene (30, 31). Although cross-resistance between RIF and RFB is common, RFB susceptibility could be predicted based on specific *rpoB* mutations in RIF-resistant isolates (30, 31). We found that the sensitivity, specificity, and concordance of RFB resistance prediction by tNGS were 94.3%, 85.7%, and 92.5%, respectively, while the agreement between tNGS and pDST showed a kappa value of 0.780 (substantial) (Table S6). The discordance between resistance in pDST and susceptibility in tNGS was 3.7% (3/81) (Table S6). One RFB-resistant isolate with an MIC of ≤0.12 μg/mL identified by pDST harbored the *rpoB* D516Y mutation, which might cause false resistance. Interestingly, the other two RFB-resistant isolates with MICs of 2 and 8 μg/mL identified by pDST harbored *rpoB* L511P/H526N double mutations. A single mutation in codons 511, 516, 526, and 533 was associated with susceptibility to RFB, while isolates with these double mutations might cause an RFB-resistant phenotype (32). The discordance between susceptibility in pDST and resistance to tNGS was 9.5% (2/21) (Table S6). Two pDST RFB-susceptible isolates harbored the high-confidence resistance mutation *rpoB* S531L, which may produce a false-susceptible result by pDST.

ETO is a structural analog of INH. Both are prodrugs that are activated by *ethA*-encoded monooxygenase and *katG*-encoded catalase-peroxidase, respectively (33). However, activated ETO and INH share the same target, the *inhA*-encoded NADH-dependent enoyl-acyl carrier protein reductase, which is involved in the long-chain mycolic acid biosynthetic pathway (33). Therefore, overexpression or modification of InhA caused by mutations in *inhA* or its promoter region results in cross-resistance to ETO and INH (34). The sensitivity, specificity, and concordance of ETO resistance prediction by tNGS were 75.0%, 97.9%, and 91.0%, respectively, while the agreement between tNGS and pDST showed a kappa value of 0.773 (substantial) (Table S6). The discordance between resistance in pDST and susceptibility in tNGS was 20.8% (5/24) (Table S6). Notably, the 5 isolates had no mutation in *inhA* or its promoter but harbored indel frameshift mutations in *ethA* detected by WGS. The discordance between susceptible in pDST and resistance in tNGS was 1.8% (1/57) (Table S6). The ETO-susceptible isolate identified by pDST harbored the low-level resistance mutation c-15t in the *inhA* promoter, which may cause a false-susceptible result by pDST (35). Since the sensitivity, specificity, and concordance of ETO resistance prediction by Deeplex Myc-TB were 95.0%, 96.6%, and 96.5%, respectively, our tNGS panel could be redesigned by adding the *ethA* gene to improve its performance (15).

Because pDST might be less reliable, most of the discordance between tNGS and pDST was in predicting EMB, PZA, CM, and KM susceptibility (35, 36). One EMB-resistant isolate with an MIC of 1 μg/mL identified by pDST had no resistance-associated mutation in *embB* or even in *embA*, *embC*, *embR*, or *ubiA*. However, one EMB-susceptible isolate identified by pDST harbored the high-confidence resistance mutation in *embB* M306V (Table 5). Two PZA-resistant isolates identified by pDST had no resistance-associated mutation in *pncA* or even in *rpoD* or *rpsA*. However, one PZA-susceptible isolate identified by pDST harbored the *pncA* H71Y mutation at a frequency of 37.1% and was detected by tNGS but not WGS and Sanger sequencing (Table 4). Three CM-resistant isolates identified by pDST had no resistance-associated mutation in *rrs* or *eis* by tNGS, but two harbored the uncharacterized novel mutation S156L or the del 357–654 frameshift mutation in *tylA* detected by WGS (37). One KM-resistant isolate identified by pDST had no resistance-associated mutation in *rrs* or *eis*. However, 2 KM-susceptible isolates identified by pDST harbored the *eis* c-12t mutation, which confers a low level of resistance to KM (Table 5) (38). One SM-resistant isolate identified by pDST had no resistance-associated mutation in *rrs*, *eis*, or *rpsL* by tNGS but harbored the uncharacterized novel mutation G71E in *gidB* detected by WGS (39). Moreover, two RIF-susceptible isolates with MICs of ≤0.12 and 0.25 μg/mL identified by pDST harbored disputed *rpoB* mutations, L511P and L533P, which caused low specificity (75%) of gDST (Table 4). Notably, one MFX-resistant isolate identified by pDST had no resistance-associated mutation in the *gyrA* or *gyrB* gene, but other mechanisms might cause resistance, such as efflux pumps (40, 41).

A tNGS assay can provide comprehensive coverage of known mutations and facilitate the discovery of uncharacterized novel or rare mutations in the full coding regions of target genes. However, the association between uncharacterized mutations and phenotypic drug resistance in M. tuberculosis warrants further study. Notably, the rare mutations *embB* V282A (EMB MIC = 4 μg/mL), *gyrB* S486F and N538T (MFX MIC = 1 and 2 μg/mL), and *Rv0678* V85F (CFZ MIC = 0.5 μg/mL) might be associated with broad phenotypic resistance to EMB, MFX, and CFZ (42), which are included in the WHO-endorsed regimens for short-course MDR-TB treatment (16). Nevertheless, tNGS can provide rapid and comprehensive DST results for timely clinical management, especially since suboptimal pDST accuracy has been found for critical first-line PZA and EMB and second-line CM and KM, which are needed for DR-TB treatment (35, 36).

Heteroresistance might be due to mixed infection with drug-resistant and drug-susceptible M. tuberculosis or with newly emerging resistant subpopulations during anti-TB drug treatment (43). Clinical samples with ≥5% minority resistant variants or with 100% resistant variants had identical pDST results (10, 44). Therefore, failure to detect minor resistant variants may lead to significant morbidity and mortality of patients and further transmission of TB. Previous studies revealed that pDST can identify an at least 1% resistant subpopulation in samples, whereas the LOD of heteroresistance for other gDST modalities was less satisfactory (44, 45). The LODs of Xpert MTB/RIF, Xpert MTB/RIF Ultra, line probe assay (LPA), and sequencing range from 20 to 90% (46–48), 5 to 40% (49), 5 to 10% (10, 44, 45), and 10 to 50% (10, 44, 45), respectively. Our results showed that tNGS is capable of detecting 2.9 to 3.8% of minority resistant variants in a heteroresistant population (Table 3). Currently, NGS has demonstrated excellent performance in providing comprehensive information for the surveillance and clinical management of DR-TB (9). In particular, tNGS captures sequence-specific regions of the genome for in-depth analyses and is more sensitive than WGS for detecting minority variants (10, 50). We found that one BDQ- and CFZ-susceptible isolate identified by pDST had a novel *Rv0678* Ins g290 to 291 frameshift mutation at a frequency of 5.6% that was detected by tNGS but not WGS or Sanger sequencing (Table 4). Since DNA samples obtained from subcultured isolates were used for WGS, a minor mutant subpopulation might be overgrown by the major wild-type subpopulation during subculture in the absence of selection pressure (51).

In this study, we demonstrated that the novel tNGS panel on the Ion AmpliSeq platform can identify numerous genome-wide targets for predicting susceptibility to 14 to 16 drugs and offers the potential to replace conventional pDST or other WHO-endorsed molecular diagnostics. In addition, our tNGS strategy is comparable to the Illumina MiniSeq WGS process and output. Currently, tNGS is streamlined and integrated into our routine TB laboratory services, with a turnaround time of 7 to 10 days, strengthening and revolutionizing the DR-TB control program.

## MATERIALS AND METHODS

### M. tuberculosis isolates.

We collected 50 M. tuberculosis isolates with various drug resistance patterns as the training set, including 46 clinical well-characterized isolates and 4 archived WHO proficiency test (WHO-PT) isolates (Table S1). For validation, 35 M. tuberculosis isolates were used as the challenge set, including 29 consecutive DR isolates from routine diagnosis services in 2022 and 6 2022 WHO-PT isolates (Table S2). This study was approved by the institutional review board of the Centers for Disease Control, Ministry of Health and Welfare (TwCDC IRB; no. 109204) and included only archived isolates; thus, written informed consent from the participants was waived. Cultivation and processing of M. tuberculosis isolates were performed in a certified biosafety level 3 laboratory. All methods were performed in accordance with the relevant guidelines and regulations.

### pDST.

M. tuberculosis isolates were subjected to phenotypic drug susceptibility testing (pDST) using the APM with 7H10 and 7H11 media (Becton, Dickinson and Company, Sparks, MD, USA). Drug resistance was defined as the growth of 1% of colonies in a drug-containing medium. According to WHO recommendations, the critical concentrations of the tested drugs in 7H10 medium were as follows: RIF, 1 μg/mL; INH, 0.2 μg/mL; EMB, 5 μg/mL; SM, 2 μg/mL; MFX, 0.25 μg/mL; and LFX, 1 μg/mL (35). The critical concentrations of the tested drugs in 7H11 medium were as follows: RFB, 0.5 μg/mL; KM, 6 μg/mL; AMK, 6 μg/mL; CM, 10 μg/mL; ETO, 10 μg/mL; *para*-aminosalicylic acid (PAS), 8.0 μg/mL; and cycloserine (CS), 60 μg/mL (35, 52). Resistance to PZA (100 μg/mL), BDQ (1 μg/mL), CFZ (1 μg/mL), LZD (1 μg/mL), and DLM (0.06 μg/mL) was tested using a Bactec MGIT 960 (Becton, Dickinson and Company) as described previously (35). The growth on the control medium was compared to that on the drug-containing medium to determine susceptibility. The DST results were categorized as indicating resistance or susceptibility, and the tests were validated by determining the susceptibility of M. tuberculosis H37Rv. MDR-TB is defined as infection with an M. tuberculosis isolate resistant to at least INH and RIF. Pre-XDR-TB is defined as infection with an MDR isolate resistant to either fluoroquinolones (FQs) (pre-XDR-FQs) or at least one of the injectable drugs (pre-XDR-INJ).

Phenotypic MIC testing for M. tuberculosis isolates was performed using Sensititre Mycobacterium tuberculosis MYCOTB plates (Thermo Scientific, TREK Diagnostic Systems, UK) or UKMYC6 plates (Thermo Scientific, UK), which are 96-well microtiter plates containing 12 (RIF, INH, EMB, SM, RFB, ofloxacin [OFX], MFX, KM, AMK, ETO, PAS, and CS) or 13 (RIF, RFB, INH, ETO, EMB, MFX, LFX, AMK, KM, BDQ, CFZ, LZD, and DLM) antimicrobial agents, respectively. The MICs were determined following the manufacturer’s instructions. The H37Rv strain was used as the control in each lot of testing, and the results were interpreted by 2 independent readers.

### Genotypic drug susceptibility testing (gDST).

**(i) Sanger sequencing.** One loopful (0.5 μL) of bacteria was placed into a microtube and resuspended in 500 μL of Tris-EDTA buffer. The bacterial solution was inactivated at 95°C for 20 min. The bacterial thermolysate was centrifuged at 12,000 × *g* for 1 min, and the supernatant was used as a template for PCR. In this study, we analyzed 22 resistance-associated genes for 14 drugs, *rpoB*, *katG*, *fabG1*, *inhA*, *embB*, *pncA*, *gyrA*, *gyrB*, *rrs*, *eis*, *rpsL*, *atpE*, *Rv0678*, *pepQ*, *Rv1979c*, *rrl*, *rplC*, *ddn*, *fgd1*, *fbiA*, *fbiB*, and *fbiC*. The PCR primers were designed based on M. tuberculosis strain H37Rv (GenBank accession no. NC_000962.3) (Table S7). PCRs were performed using a HotStarTaq master mix kit (Qiagen GmbH, Hilden, Germany). Each reaction mixture contained 12.5 μL of 2× HotStarTaq master mix (Qiagen), 0.5 μL of each primer (10 μM), and 2 to 5 μL of bacterial lysate. Double-distilled water was added to the mixture to obtain a total volume of 25 μL. The PCR conditions were as follows: hot start at 95°C for 10 min; 35 cycles of 95°C for 1 min, 55 to 66°C (according to the optimal primer annealing temperature) for 1 min, and 72°C for 1 min; and a final elongation step of 72°C for 5 min. The PCR products were analyzed using a capillary electrophoresis QIAxcel Advanced system (Qiagen). The DNA sequence was confirmed by Sanger sequencing (Genomics BioSci & Tech, Taiwan). In addition, sequence assembly and mutation identification were performed using Sequencher (Gene Codes Corporation, USA) and Molecular Evolutionary Genetics Analysis 10 (MEGA 10) software.

**(ii) Targeted NGS.** Bacterial thermolysate was used for multiplex PCR of an Ion AmpliSeq custom panel covering the target regions of *rpoB*, *katG*, *fabG1*, *inhA*, *embB*, *pncA*, *gyrA*, *gyrB*, *rrs*, *eis*, *rpsL*, *atpE*, *Rv0678*, *pepQ*, *Rv1979c*, *rrl*, *rplC*, *ddn*, *fgd1*, *fbiA*, *fbiB*, and *fbiC* (Thermo Fisher Scientific, Waltham, MA, USA), including 329 amplicons (Table 2). The quantity of nucleic acid in bacterial lysates was estimated by a Qubit double-stranded DNA (dsDNA) highly selective (HS) assay kit and a Qubit One fluorometer (Thermo Fisher Scientific). Library construction of the amplicons was performed according to the protocol of Ion AmpliSeq Library Kits 2.0 (Thermo Fisher Scientific). The subsequent preparation and enrichment of the sequencing beads were performed according to the protocol of the Ion Chef kit (Thermo Fisher Scientific). The barcodes used for each sample were as follows. Sequencing was performed on a 520 chip using an Ion GeneStudio S5 Prime (Thermo Fisher Scientific) according to the protocol of the Ion 510/520/530-Chef kit. Base calling was performed by using built-in Torrent Suite v5.10.0 software (Thermo Fisher Scientific). Variant calling was performed using VariantCaller v5.10.0 (Thermo Fisher Scientific). The variant allele frequency (VAF) was defined as the percentage of mutant reads at a particular locus. The antimicrobial resistance predictions are based on *Catalogue of Mutations in* Mycobacterium tuberculosis *Complex and Their Association with Drug Resistance*, issued by the WHO (17).

**(iii) Whole-genome sequencing.** Genomic DNA was extracted following the phenol-chloroform method and quantified using a Qubit 4.0 fluorometer (Thermo Fisher Scientific, Waltham, MA, USA). Paired-end libraries were prepared using a TruSeq DNA PCR-free high-throughput (HT) sample preparation kit (Illumina, Inc., San Diego, CA, USA) according to the manufacturer’s protocol. The average fragment size (500 to 600 bp) of the DNA libraries was estimated by an Agilent 2100 Bioanalyzer. The 24 purified DNA libraries were pooled, and the DNA concentration was quantified with a Qubit 4.0 fluorometer. The pooled libraries (11 pM) were sequenced on an Illumina MiSeq system (Illumina, Inc.) with a MiSeq reagent kit version 3 (600 cycles), which showed that the first paired-end reads were 350 nucleotides (nt) in length, whereas the second paired-end reads were 250 nt in length. Sequencing reads were checked using fastQC (www.bioinformatics.babraham.ac.uk/projects/fastqc/) as a primary assessment of data quality and then analyzed using the TB-Profiler tool for drug resistance prediction (53).

### Detection of heteroresistance.

The pansusceptible H37Rv isolate and a well-characterized drug-resistant isolate with *rpoB* S531L, *katG* S315T, *embB* D328Y, *pncA* V115G, and *gyrA* D94A mutations were used to determine the limit of heteroresistance detection. We transferred H37Rv colonies obtained from 7H11 agar to a sterile tube containing glass beads and quantified the bacterial mass using a calibrated precision balance. These bacteria were resuspended in saline and homogenized by vortex. The resistant and susceptible isolates were adjusted to MacFarland 0.5 mg/mL and were mixed in the following proportions of resistant to susceptible organisms: 100:0, 32:68, 16:84, 8:92, 4:96, 2:98, 1:99, and 0:100. Each mixture was checked in triplicate by our tNGS assay. The LOD is defined in this study as the lowest VAF that can be detected in triplicates.

### Statistical analyses.

The sensitivity, specificity, concordance, and agreement were calculated excluding isolates with uncharacterized mutations, including novel and rare variants. The agreement between phenotypic and genotypic DST results was evaluated by kappa and 95% confidence interval (CI) values. The kappa result was interpreted as follows: values of 0.81 to 1.00 indicated almost perfect agreement, values of 0.61 to 0.80 indicated substantial agreement, values of 0.41 to 0.60 indicated moderate agreement, values of 0.21 to 0.40 indicated fair agreement, and values of ≤0.20 indicated slight to no agreement (54).

### Data availability.

Sequencing reads obtained from Ion Torrent for tNGS (BioProject no. PRJNA884755) and Illumina for WGS (BioProject no. PRJNA884747) have been deposited in the NCBI server.
