# Supplementary material for: Development and Assessment of a Novel Whole-Gene-Based Targeted Next-Generation Sequencing Assay for Detecting the Susceptibility of Mycobacterium tuberculosis to 14 Drugs
Source: Microbiol Spectr. 2022 Oct 18;10(6):e02605-22. doi: 10.1128/spectrum.02605-22 (PMC9769975; doi:10.1128/spectrum.02605-22)
Supplement: Supplemental file 1 — Supplemental material. Download spectrum.02605-22-s0001.pdf, PDF file, 0.3 MB [file spectrum.02605-22-s0001.pdf]

Supplementary Material

TABLE S1 The drug resistance profile of the training isolates (N=50)

**Drug resistance profile**	**No. (%) of isolates**
**Pansusceptible**	2 (4)
**Rifampicin resistant**	7 (14)
RIF	3
RIF+EMB	1
RIF+PZA	1
RIF+BDQ+CFZ	2
**Multidrug resistant**	18 (36)
RIF+INH	7
RIF+INH+EMB	2
RIF+INH+SM	1
RIF+INH+EMB+SM	1
RIF+INH+SM+BDQ	1
RIF+INH+EMB+PZA+SM	2
RIF+INH+EMB+BDQ+CFZ	1
RIF+INH+PZA+SM+DLM	1
RIF+INH+EMB+PZA+SM+BDQ+CFZ	2
**Pre-extensively drug resistant**	14 (28)
RIF+INH+KM	1
RIF+INH+MFX+LFX	1
RIF+INH+EMB+PZA+CM	1
RIF+INH+PZA+CM+SM	1
RIF+INH+AMK+CM+KM	1
RIF+INH+EMB+MFX+LFX	1
RIF+INH+EMB+MFX+SM	1
RIF+INH+EMB+MFX+LFX+SM	2
RIF+INH+EMB+AMK+CM+KM+SM	1
RIF+INH+MFX+LFX+SM+BDQ+CFZ	1
RIF+INH+EMB+PZA+MFX+LFX+SM	2
RIF+INH+EMB+PZA+MFX+LFX+DLM	1
**Extensively drug resistant**	3 (6)
RIF+INH+EMB+MFX+LFX+AMK+CM+KM	1
RIF+INH+EMB+PZA+MFX+LFX+SM+BDQ+CFZ	1
RIF+INH+EMB+PZA+MFX+LFX+AMK+CM+KM+SM	1
**Others**	6 (12)
BDQ	1*
LZD	1*
INH+SM	1
INH+DLM	1*
INH+EMB+SM	1
AMK+CM+SM+BDQ+CFZ	1*

RIF, rifampicin; INH, isoniazid; EMB, ethambutol; PZA, pyrazinamide; MFX, moxifloxacin; LFX, levofloxacin; AMK, amikacin; CM, capreomycin; KM, kanamycin; SM, streptomycin; BDQ, bedaquiline; CFZ, clofazimine; LZD, linezolid; DLM, delamanid. * WHO proficiency test isolates.

TABLE S2 The drug resistance profile of the challenge isolates (N=35)

Drug resistance profile	No. (%) of isolates
**Rifampicin resistant**	2 (5.7)
RIF	1
RIF+KM	1
**Multidrug resistant**	21 (60.0)
RIF+INH	7
RIF+INH+EMB	2
RIF+INH+SM	1
RIF+INH+EMB+PZA	1
RIF+INH+EMB+SM	7
RIF+INH+PZA+SM	1
RIF+INH+BDQ+CFZ	1
RIF+INH+EMB+PZA+SM	1
**Pre-extensively drug resistant**	5 (14.3)
RIF+INH+EMB+MFX+LFX	1
RIF+INH+AMK+CM+KM+SM	1
RIF+INH+EMB+PZA+MFX+LFX+SM	1
RIF+INH+EMB+PZA+AMK+CM+KM+SM	1*
RIF+INH+EMB+PZA+MFX+LFX+SM+BDQ+LZD	1*
**Others**	7 (20.0)
INH	1
BDQ	1*
LZD	1*
INH+CM	1
MFX+LFX	1*
BDQ+CFZ	1*
INH+EMB+PZA	1

RIF, rifampicin; INH, isoniazid; EMB, ethambutol; PZA, pyrazinamide; MFX, moxifloxacin; LFX, levofloxacin; AMK, amikacin; CM, capreomycin; KM, kanamycin; SM, streptomycin; BDQ, bedaquiline; CFZ, clofazimine; LZD, linezolid; DLM, delamanid. * WHO proficiency test isolates.

TABLE S5 Catalog of uncharacterized novel (*) or rare (#) variants identified by tNGS

Drug	Uncharacterized variants (variant allele frequency)	pDST	
		R	S
RIF	*rpoB* H526A*	0	1
	rpoB H531V#	0	1
INH	*katG* Del 35-42 Fs*	1	0
	katG L148Q*	1	0
	katG T271I#	1	0
	katG Y304D*	1	0
	katG G307E#/*fabG1* g-17t#	1	0
	katG Y413C#	1	0
	katG G712D*	1	0
	fabG1 promoter Del	1	0
	katG L101P*/S140N#	0	1
	katG L458C*	0	1
	fabG1 L91V*	0	1
	fabG1 S126N#	0	1
EMB	*embB* g-6a*	1	0
	embB N399T#	1	0
	embB L402V#/S565G#	1	0
	embB E405D#	1	0
	embB Q497P#/D1024N#	3	0
	embB D814N*	1	1
	embB H1002R#	1	0
	embB G175A*	0	1
	embB G246R#	0	2
	embB G246R#/Y315C#/D1024N#	0	1
	embB V282A#	0	1
	embB D354N#	0	1
PZA	*pncA* H51P#	1	0
	pncA D136N#	1	0
	pncA V163A#	1	0
	pncA S67L*	0	1
MFX/LFX	*gyrA* A210V#	0	1
	gyrA R252G#/N826S*	0	1
	gyrB S486F#	0	1
	gyrB N538T#	0	1
	gyrB G551R#	0	1
	gyrB A680Del#	0	1
AMK/CM/KM	*rrs* a13g#	0	1
	rrs t16c#	0	2
	rrs g336a*	0	1
	rrs c594t*	0	1
	rrs Ins t 1206-1207*	0	1

	eis A86T* (22.4 or 29.6%)/V87L* (22.4 or 29.6%)	0	2
	eis Del 805-806 Fs*	0	1
SM	*rrs* a13g#	1	0
	rrs t16c#	2	0
	rrs g336a*	0	1
	rrs c594t*	1	0
	rrs Ins t 1206-1207*	1	0
BDQ	*Rv0678* whole gene Del#	2	0
	Rv0678 Del 11-63 Fs# (44.4%)	2	0
	Rv0678 G25S* (21.8%)/Ins g 181-182 Fs# (83.1%)	1	0
	Rv0678 G41V*	1	0
	Rv0678 C46Y#	1	0
	Rv0678 S53P#	1	0
	Rv0678 T69P*	1	0
	Rv0678 V85F#	1	0
	Rv0678 R90C #/*pepQ* S320F*	1	0
	Rv0678 Ins g 418-419 Fs#	1	0
	Rv0678 D5A*	0	1
	Rv0678 Ins g 290-291 Fs* (5.6%)	0	1
	Rv0678 V120M#	0	1
CFZ	*Rv0678* whole gene Del#	2	0
	Rv0678 Del 11-63 Fs#	2	0
	Rv0678 G25S* (21.8%)/Ins g 181-182 Fs# (83.1%)	1	0
	Rv0678 G41V*	1	0
	Rv0678 C46Y#	1	0
	Rv0678 S53P#	1	0
	Rv0678 T69P*	1	0
	Rv0678 D5A*	0	1
	Rv0678 V85F#	0	1
	Rv0678 R90C #/*pepQ* S320F*	0	1
	Rv0678 Ins g 290-291 Fs* (5.6%)	0	1
	Rv0678 V120M#	0	1
	Rv0678 Ins g 418-419 Fs#	0	1
	Rv1979c V52G#/A354T*	0	2
	Rv1979c S448A*	0	1
	Rv1979c Y467H*	0	1
LZD	*rrl* t100c*	0	1
	rrl g249a*	0	1
	rrl t896c#	0	1
	rrl a1075g#	0	4
	rrl a2107g#	0	1
	rrl c2341a#	0	2
	rrl c2341a#/t3023a*	0	1
	rrl Ins a 2354-2355*	0	1

	rrl c2382t[*]	0	1
	rrl g2466t[*]	0	1
	rrl a3136c[*]	0	1
DLM	*ddn* W27stop[#]	1	0
	ddn P154R[*]*/fgd1* Ins 5 bp 35-36 Fs[*] (14.9%)/*fbiB* P361R[*] (5.2%)	1	0
	fgd1 Ins 5 bp 35-36 Fs[*] (12.2 or 17.2%)	1	1
	fgd1 K270M[#]	0	2
	fgd1 Ins 5 bp 35-36 Fs[*] (15.3%)/*fbiB* P361R[*] (10.8%)	0	1
	fgd1 Ins 5 bp 35-36 Fs[*] (16.4%)/*fbiC* A505T[#]	0	1
	fbiA R175H[#]	0	1
	fbiA V188I[#]	0	1
	fbiB P361R[*] (6.7 or 8.1%)	0	2
	fbiC V16I[#]/G232R[*]	0	1
	fbiC A505T[#]	0	8

RIF, rifampicin; INH, isoniazid; EMB, ethambutol; PZA, pyrazinamide; MFX, moxifloxacin; LFX, levofloxacin; AMK, amikacin; CM, capreomycin; KM, kanamycin; SM, streptomycin; BDQ, bedaquiline; CFZ, clofazimine; LZD, linezolid; DLM, delamanid; pDST, phenotypic drug susceptibility testing; R, resistant; S, susceptible; Ins, insertion; Del, deletion; Fs, frameshift mutation; [*], novel mutation; [#], rare mutation.

TABLE S6 Performance of gDST on the training and challenge isolates (N=81*)

Drug	Method	pDST resistant				pDST susceptible				Performance (excluding uncharacterized variants)			
		gDST (N)			Total (N)	gDST (N)			Total (N)	Sensitivity %	Specificity %	Concordance %	Agreement kappa (95% confidence interval)
		R	S	U		R	S	U					
RFB	tNGS	50	3	7	60	2	12	7	21	94.3	85.7	92.5	0.780 (0.596-0.964)
	WGS	50	3	7	60	2	12	7	21	94.3	85.7	92.5	0.780 (0.596-0.964)
	Sanger	50	3	7	60	2	12	7	21	94.3	85.7	92.5	0.780 (0.596-0.964)
ETH	tNGS	15	5	4	24	1	46	10	57	75.0	97.9	91.0	0.773 (0.602-0.944)
	WGS	15	0	9	24	1	46	10	57	100.0	97.9	98.4	0.957 (0.874-1.000)
	Sanger	15	5	4	24	1	46	10	57	75.0	97.9	91.0	0.773 (0.602-0.944)

pDST, phenotypic drug susceptibility testing; gDST, genotypic drug susceptibility testing; Sanger, Sanger sequencing; tNGS, targeted NGS; WGS, whole-genome sequencing; R: detection of resistance-associated mutation; S: detection of mutations known to not be associated with resistance (phylogenetic marker or synonymous mutation) or no mutation detected; U: detection of at least one novel nonsynonymous mutation; NA, not available. *Excluding 4 isolates that had no pDST result for RFB and ETO.

TABLE S7 Drug resistance genes, primer information and targeted regions of *Mycobacterium tuberculosis* complex for Sanger sequencing

Targets	Primer names	Sequences (5′→3′)	Genome positions	Gene positions	Codons
rpoB	*rpoB*-1F	CTTCTCCGGGTCGATGTCGTTG	760082-760103	276-640	93-213
	rpoB-1R	CGCGCTTGTCGACGTCAAACTC	760425-760446		
	rpoB-2F	TCGGCGAGCCCATCACGTCG	760759-760778	953-1550	319-516
	rpoB-2R	ACGCCGTCGACCACCTTGCGGTA	761334-761356		
katG	*katG*-F	GTTGAATGACTCCTGGATCTCTTC	2154525-2154548	635-1587	213-529
	katG-R	GTAAGCGGGATCTGGAGAACC	2155457-2155477		
fabG1_inhA	*fabG1*-F	TGCTGAGTCACACCGACAAAC	1673310-1673330	*fabG1* (-131)-*inhA* 84	(-131)-*fabG1* full CDS-*inhA* 28
	inhA-R	TACCCGTGCGATGTGAAACG	1674266-1674285		
embB	*embB*-F	CGACGCCGTGGTGATATTCG	4247350-4247369	837-1699	280-566
	embB-R	CCACGCTGGGAATTCGCTTG	4248193-4248212		
pncA	*pncA*-F	GCTGGTCATGTTCGCGATCG	2289326-2289345	(-105)-(+57)	(-105)-Full CDS
	pncA-R	CGCTTGCGGCGAGCGCTCCA	2288625-2288644		
gyrA	*gyrA*-F	GATGACAGACACGACGTTGC	7301-7320	(-2)-425	(-2)-141
	gyrA-R	AGCATCTCCATCGCCAACG	7708-7726		
gyrB	*gyrB*-F	AAGACCAAGTTGGGCAACAC	6353-6372	1114-1722	373-574
	gyrB-R	CTGCCACTTGAGTTTGTACA	6942-6961		
rrs	*rrs*-F	GCGATGCCGCGAGGTTAAGC	1473095-1473114	1249-1567	NA
	rrs-R	ACCAGTTGGGGCGTTTTCGT	1473393-1473412		
eis	*eis*-F	ATTCAGGGCCGATGAAATC	2715239-2715257	(-366)-(+94)	(-366)-31
	eis-R	GATGATCGACCGGGTTTG	2715680-2715697		
rpsL	*rpsL*-F	CGCGATGCCTCGGATGAGACG	781434-781454	(-126)-(+79)	(-126)-Full CDS
	rpsL-R	ACCAACTGGGTGACCAACTGCG	781992-782013		
atpE	*atpE*-F	CCAAGCGATGGAGCTCGAAGAGG	1460963-1460985	(-83)-(+112)	(-83)-Full CDS
	atpE-R	GGGAATGAGGAAGTTGCTGGACTCG	1461377-1461401		
Rv0678	*Rv0678*-F	GCTTGAGAGTTCCAATCAT	778887-778905	(-104)-(+74)	(-104)-Full CDS
	Rv0678-R	CGCATCAACAAGGAGTGA	779543-779560		
pepQ	*pepQ*-1F	GAACAGGCGGAGAACCACCATCG	2860492-2860514	(-75)-(+106)	(-75)-Full CDS
	pepQ-1R	GGCGCCGAAGTCGATCTTCACG	2859747-2859768		
	pepQ-2F	TGATGCTCGATCATGGCGCTGACG	2859860-2859883		
	pepQ-2R	CTTGCCCGGTTTGACGTGCTGG	2859195-2859216		
rrl	*rrl*-F	GCAAGGGTGAAGCGGAGAA	1475762-1475780	2105-3005	NA
	rrl-R	GTTTCCCGCTTAGATGCTTTCAG	1476640-1476662		
rplC	*rplC*-F	ACCGCATAAGTACAAGGACTCG	800648-800669	(-162)-(+112)	(-162)-Full CDS
	rplC-R	CGATGTTGGCCGGGACGTC	801555-801573		
Rv1979c	*Rv1979c*-1F	CAGGCCGGGAGCTCAAGAATCG	2223220-2223241	(-78)-(+101)	(-78)-Full CDS
	Rv1979c-1R	GCATCGCTACCGTTAGGGTGAGC	2222339-2222361		
	Rv1979c-2F	CGAACGCCGCCGAAGAAATGG	2222429-2222449		
	Rv1979c-2R	CCGATCCGGGAATGCAGGTTG	2221617-2221637		
ddn	*ddn*-F	CCACCGGCACCATCATCGAGCG	3986704-3986725	(-141)-(+137)	(-141)-Full CDS
	ddn-R	GTCAGCGACCTGTTGGCCAAGTCG	3987412-3987435		
fgd1	*fgd1*-1F	GCCGCGAGCGAGGTGAACC	490598-490616	(-185)-(+56)	(-185)-Full CDS
	fgd1-1R	CCCCGTCGGGCACGTCGTAGATCG	491264-491287		

	fgd1-2F	TTCGCCCGGCTGCGTGAATCG	491167-491187		
	fgd1-2R	AATCGTCGACTTACCCGTCTGCG	491826-491848		
fbiA_fbiB	*fbiA*-F	GGCGTGTCGAGCGTGACTCG	3640478-3640497		
	fbiA-R	CGCCGTCGTGCACCAGCCAGC	3641402-3641422		
	fbiA/B-F	TGCGCGGCATGGCCGATACG	3641297-3641316		
	fbiA/B-R	GCAGCAGTTGCCGGGCTGTCGAGC	3642211-3642234	(-65)-(+84)	(-65)-Full CDS
	fbiB-F	CATACGGCAATGAGTTGGTGGTCACC	3642079-3642104		
	fbiB-R	GAATTGATCCGTCGGGAGGTTGATGG	3642939-3642964		
fbiC	*fbiC*-1F	GGTGCCGGACGGTCGACGG	1302801-1302819		
	fbiC-1R	CACTTCTTGGATATGCCCGAACTCC	1303776-1303800		
	fbiC-2F	GCATCGGCGAGACGCTATCCG	1303715-1303735		
	fbiC-2R	CCGACCGACAGCGAGTAGGCG	1304655-1304675	(-130)-(+70)	(-130)-Full CDS
	fbiC-3F	TCAACTTCACCAACATCTGCTACACCG	1304585-1304611		
	fbiC-3R	GCCGGGGCTAGGCCAGATTGC	1305550-1305570		

CDS, coding sequence, NA: not applicable for codons (positions outside a CDS or in rDNA regions). Positions of the reference sequences relative to the genome and genes of the *M. tuberculosis* H37Rv strain are indicated. Gene positions in promoter or 3' regions relative to the +1 or last nucleotide of CDSs are indicated by minus (-) or plus (+) signs, respectively.
